# Updated checklist of Azores Chondrichthyes (Vertebrata: Gnathostomata)

**DOI:** 10.3897/BDJ.9.e62813

**Published:** 2021-03-11

**Authors:** Luís M. D. Barcelos, José M. N. Azevedo, João Pedro Barreiros

**Affiliations:** 1 University of the Azores, Angra do Heroísmo, Portugal University of the Azores Angra do Heroísmo Portugal; 2 Centre for Ecology, Evolution and Environmental Changes (cE3c), Angra do Heroísmo, Portugal Centre for Ecology, Evolution and Environmental Changes (cE3c) Angra do Heroísmo Portugal; 3 Centre for Ecology, Evolution and Environmental Changes (cE3c), Ponta Delgada, Portugal Centre for Ecology, Evolution and Environmental Changes (cE3c) Ponta Delgada Portugal

**Keywords:** cartilaginous fishes, rays, sharks, species list

## Abstract

**Background:**

Several lists of marine fish from Azores have been published in the past. Most of those publications are difficult to access on line and several were not published in peer-reviewed journals.

**New information:**

This checklist updates all the chondrichthyan records for the Azores Exclusive Economic Zone (EEZ), according to the most recent taxonomic classification of cartilaginous fish, as well as providing information on the conservation status for all species. We also present recent literature data on rare species and recent records for Azores. This is the first comprehensive list of cartilaginous fishes from Azores to be published in the GBIF database.

## Introduction

Chondrichthyes are commonly known as cartilaginous fishes and includes chimeras, sharks and rays. They can be found from the cold deep-sea to subtropical and tropical waters (Greemberg 2009). Sharks and rays have a great recreational and ecological interest (Vieira et al. 2020); however, many species worldwide are declining due to global overfishing (Worm et al. 2013). The total estimation for risk extinction, according to the IUCN Red List criteria, indicates that a quarter of all shark and ray species are threatened (Dulvy et al. 2014).

The first comprehensive chondrichthyan checklist for Azores was published over 20 years ago by Santos et al. (1997). After that, some updates were made, most of them integrated in marine fish or biodiversity lists (e.g. Porteiro et al. 2010). In the meantime, new publications have emerged regarding new records (e.g. Catarino et al. 2020) or with new information on species already recorded for Azores (e.g. Das and Afonso 2017, Barcelos et al. 2018). The present work gives taxonomic updates and information regarding the conservation status (IUCN) for all chondrichthyan species from the Azores' EEZ (see Suppl. material 1). This is the first complete and updated list of chondrichthyans that can be found in the GBIF online platform (Barcelos et al. 2020).

## General description

### Purpose

Updating Azorean biota checklists.

## Project description

### Title

Azores Chondrichthyes updated checklist

### Personnel

Paulo Borges

### Study area description

Azores Archipelago

### Funding

Funding Institutions: Azores PO 2020 - ACORES-01-0145-FEDER-000072; TOTAL BUDGET: €299,901.83 EU Support: €254,916.56. This project was financed by FEDER in 85% and by Azorean Public funds by 15% through the Operational Programme Azores 2020. This work is also funded by FEDER funds through the COMPETE 2020 Programme and National Funds through FCT - Portuguese Foundation for Science and Technology under the Research Infrastructure PORBIOTA - Portuguese E-Infrastructure for Information and Research on Biodiversity, project number POCI-01-0145-FEDER-022127.

## Geographic coverage

### Description

The Azores EEZ (Exclusive Economic Zone).

### Coordinates

34 and 42.976 Latitude; -35.578 and -21 Longitude.

## Taxonomic coverage

### Description

This dataset covers all the sharks, rays and chimaeras so far known to occur within the Azores' EEZ.

### Taxa included

**Table:** 

Rank	Scientific Name	
kingdom	Animalia	
phylum	Chordata	
class	Chondrichthyes	
subclass	Holocephali	
subclass	Elasmobranchii	
superorder	Holocephalimorpha	
superorder	Selachimorfa	
superorder	Batoidea	
order	Carcharhiniformes	Ground sharks
order	Chimaeriformes	Chimaeras
order	Hexanchiformes	Frill and cow sharks
order	Lamniformes	Mackerel sharks
order	Myliobatiformes	Stingrays
order	Orectolobiformes	Carpet sharks
order	Rajiformes	Skates and rays
order	Squaliformes	Bramble, sleeper and dogfish sharks
order	Torpediniformes	Electric rays
family	Rhinochimaeridae	
family	Chimaeridae	
family	Sphyrnidae	
family	Carcharhinidae	
family	Triakidae	
family	Pseudotriakidae	
family	Scyliorhinidae	
family	Pentanchidae	
family	Lamnidae	
family	Cetorhinidae	
family	Alopiidae	
family	Odontaspididae	
family	Rhincodontidae	
family	Dalatiidae	
family	Oxynotidae	
family	Somniosidae	
family	Etmopteridae	
family	Centrophoridae	
family	Hexanchidae	
family	Chlamydoselachidae	
family	Mobulidae	
family	Myliobatidae	
family	Dasyatidae	
family	Rajidae	
family	Torpedinidae	
species	*Rhinochimaeraatlantica* Holt & Byrne, 1909	
species	*Hydrolaguspallidus* Hardy & Stehmann, 1990	
species	*Hydrolagusaffinis* (de Brito Capello, 1868)	
species	*Chimaeraopalescens* Luchetti, Iglésias & Sellos, 2011	
species	*Chimaeramonstrosa* Linnaeus, 1758	
species	*Sphyrnazygaena* (Linnaeus, 1758)	
species	*Prionaceglauca* (Linnaeus, 1758)	
species	*Galeocerdocuvier* (Péron & Lesueur, 1822)	
species	*Carcharhinuslongimanus* (Poey, 1861)	
species	*Carcharhinusleucas* (Valenciennes, 1839)	
species	*Carcharhinusgalapagensis* (Snodgrass & Heller, 1905)	
species	*Galeorhinusgaleus* (Linnaeus, 1758)	
species	*Pseudotriakismicrodon* de Brito Capello, 1868	
species	*Scyliorhinuscanicula* (Linnaeus, 1758)	
species	*Galeusmurinus* (Collett, 1904)	
species	*Apristurusmanis* (Springer 1979)	
species	*Apristuruslaurussonii* (Saemundsson, 1922)	
species	*Lamnanasus* (Bonnaterre, 1788)	
species	*Isuruspaucus* Guitart Manday, 1966	
species	*Isurusoxyrinchus* Rafinesque, 1810	
species	*Carcharodoncarcharias* (Linnaeus, 1758)	
species	*Cetorhinusmaximus* (Gunnerus, 1765)	
species	*Alopiasvulpinus* (Bonnaterre, 1788)	
species	*Alopiassuperciliosus* (Lowe, 1841)	
species	*Odontaspisferox* (Risso, 1810)	
species	*Rhincodontypus* Smith, 1828	
species	*Squalioluslaticaudus* Smith & Radcliffe, 1912	
species	*Dalatiaslicha* (Bonnaterre, 1788)	
species	*Oxynotusparadoxus* Frade, 1929	
species	*Zameussquamulosus* (Günther, 1877)	
species	*Somniosusrostratus* (Risso, 1827)	
species	*Somniosusmicrocephalus* (Bloch & Schneider, 1801)	
species	*Scymnodalatiasgarricki* Kukuev & Konovalenko, 1988	
species	*Centroselachuscrepidater* (Barbosa du Bocage & de Brito Capello, 1864)	
species	*Centroscymnusowstonii* Garman, 1906	
species	*Centroscymnuscoelolepis* Barbosa du Bocage & de Brito Capello, 1864	
species	*Etmopterusspinax* (Linnaeus, 1758)	
species	*Etmopteruspusillus* (Lowe, 1839)	
species	*Etmopterusprinceps* Collett, 1904	
species	*Centroscylliumfabricii* (Reinhardt, 1825)	
species	*Deaniaprofundorum* (Smith & Radcliffe, 1912)	
species	*Deaniacalcea* (Lowe, 1839)	
species	*Centrophorussquamosus* (Bonnaterre, 1788)	
species	*Centrophorusgranulosus* (Bloch & Schneider, 1801)	
species	*Hexanchusgriseus* (Bonnaterre, 1788)	
species	*Heptranchiasperlo* (Bonnaterre, 1788)	
species	*Chlamydoselachusanguineus* Garman, 1884	
species	*Mobulatarapacana* (Philippi, 1892)	
species	*Mobulamobular* (Bonnaterre, 1788)	
species	*Mobulabirostris* (Walbaum, 1792)	
species	*Myliobatisaquila* (Linnaeus, 1758)	
species	*Dasyatispastinaca* (Linnaeus, 1758)	
species	*Pteroplatytrygonviolacea* (Bonaparte, 1832)	
species	*Taeniuropsgrabatus* (Geoffroy St. Hilaire, 1817)	
species	*Dipturusbatis* (Linnaeus, 1758)	
species	*Leucorajafullonica* (Linnaeus, 1758)	
species	*Rajabrachyura* Lafont, 1873	
species	*Rajaclavata* Linnaeus, 1758	
species	*Rajamaderensis* Lowe, 1838	
species	*Rajellabigelowi* (Stehmann, 1978)	
species	*Dipturusoxyrinchus* (Linnaeus, 1758)	
species	*Bathyrajarichardsoni* (Garrick, 1961)	
species	*Tetronarcenobiliana* (Bonaparte, 1835)	

## Usage licence

### Usage licence

Creative Commons Public Domain Waiver (CC-Zero)

### IP rights notes

This work is licensed under a Creative Commons Attribution (CC-BY) 4.0 Licence.

## Data resources

### Data package title

Azores chondrichthyes updated checklist

### Resource link

http://ipt.gbif.pt/ipt/resource?r=azores_chondrichthyes_updated_checklist_vs_1&v=1.9

### Alternative identifiers

http://ipt.gbif.pt/ipt/resource?r=azores_chondrichthyes_updated_checklist_vs_1

### Number of data sets

1

### Data set 1.

#### Data set name

Azores chondrichthyes updated checklist

#### Data format

Darwin Core

#### Description

This updated checklist of the chondrichthyans already identified within the Azores EEZ follows the last revised version published by Barreiros and Gadig (2011). Two new records have been registered and the taxonomy has been updated. New information regarding the specific occurrence of the rare *Odontaspisferox* was recently published (see Barcelos et al. 2018). The first comprehensive chondrichthyan checklist was published by Santos et al. (1997) and later updated by Porteiro et al. (2010).

**Data set 1.** 

Column label	Column description
id	identification of the record
taxonID	identification of the taxon
acceptedNameUsageID	identification of the usage name
parentNameUsageID	identifiction of the parent usage name
scientificName	scientific name of the taxon
parentNameUsage	name from which the taxon name originates
kingdom	kingdom
phylum	phylum
class	class
order	order
family	family
genus	genus
specificEpithet	specific epithet
taxonRank	taxon rank
scientificNameAuthorship	scientific name authorship

## Additional information

The following species are referred to the Azores’ EEZ by some authors: *Bathytoshiacentroura* (Das and Afonso 2017, Santos et al. 2020), *Deaniahystricosa* (Compagno 2016), *Dipturusintermedius* (Santos et al. 2020) and *Bathyrajapallida* (Das and Afonso 2017, Santos et al. 2020). However, they were not included in this checklist because those authors, as well as other sources (e.g. IUCN’s Red List) report their occurrence as either doubtful or ‘need to be confirmed’. As such, this fact does not allow us to have a degree of confidence that these species were actually confirmed to occur within this checklist geographic area.

## Supplementary Material

90267D5A-DACF-5E92-B140-A0443022674F 10.3897/BDJ.9.e62813.suppl1 Supplementary material 1 Species list - taxon remarks and occurrence details Data type taxon remarks, occurrence details, IUCN conservation status File: oo_510738.xlsx https://binary.pensoft.net/file/510738 Barcelos LMD, Azevedo JMN, Barreiros JP
